# Striatal Neurons Partially Expressing a Dopaminergic Phenotype: Functional Significance and Regulation

**DOI:** 10.3390/ijms231911054

**Published:** 2022-09-21

**Authors:** Dmitry Troshev, Alyona Bannikova, Victor Blokhin, Anna Kolacheva, Tatiana Pronina, Michael Ugrumov

**Affiliations:** Laboratory of Neural and Neuroendocrine Regulations, Koltzov Institute of Developmental Biology of the Russian Academy of Sciences, 119334 Moscow, Russia

**Keywords:** dorsal striatum, dopamine, tyrosine hydroxylase, aromatic l-amino acid decarboxylase, green fluorescent protein, neuronal regulation, fluorescence-activated cell sorting, laser microdissection, transgenic mice

## Abstract

Since the discovery of striatal neurons expressing dopamine-synthesizing enzymes, researchers have attempted to identify their phenotype and functional significance. In this study, it was shown that in transgenic mice expressing green fluorescent protein (GFP) under the tyrosine hydroxylase (TH) gene promoter, (i) there are striatal neurons expressing only TH, only aromatic L-amino acid decarboxylase (AADC), or both enzymes of dopamine synthesis; (ii) striatal neurons expressing dopamine-synthesizing enzymes are not dopaminergic since they lack a dopamine transporter; (iii) monoenzymatic neurons expressing individual complementary dopamine-synthesizing enzymes produce this neurotransmitter in cooperation; (iv) striatal nerve fibers containing only TH, only AADC, or both enzymes project into the lateral ventricles, providing delivery pathways for L-3,4-dihydroxyphenylalanine and dopamine to the cerebrospinal fluid; and (v) striatal GFP neurons express receptor genes for various signaling molecules, i.e., classical neurotransmitters, neuropeptides, and steroids, indicating fine regulation of these neurons. Based on our data, it is assumed that the synthesis of dopamine by striatal neurons is a compensatory response to the death of nigral dopaminergic neurons in Parkinson’s disease, which opens broad prospects for the development of a fundamentally novel antiparkinsonian therapy.

## 1. Introduction

Nigrostriatal dopaminergic neurons of the brain play a key role in the regulation of motor behavior, motor memory, and some other types of behavior [1]. The degeneration of these neurons and the resulting dopamine (DA) deficiency in the striatum lead to the development of Parkinson’s disease [2,3,4,5]. Until the end of the 1980s, there was no doubt that the only source of DA in the striatum is the axons of DAergic neurons located in the substantia nigra. However, later, immunocytochemical studies showed that the striatum contains neurons expressing tyrosine hydroxylase (TH), the first rate-limiting enzyme of DA synthesis [6,7,8,9,10,11,12,13]. This raised the question of whether these neurons are DAergic, synthesizing DA.

Over the past two decades, it has been shown using immunocytochemistry that TH-expressing neurons are an attribute of the striatum in all studied mammals, including non-human primates and humans [11,12]. Moreover, it turned out that the number of TH neurons in the striatum is species-specific. In rodents, the striatum contains rare neurons, while in primates (monkeys and humans), it contains hundreds or even thousands of such neurons [6,7,9,10,12].

Of particular interest is the fact that the number of TH neurons increases significantly after DAergic denervation of the striatum. This is also the case for patients with Parkinson’s disease [11,12,14,15,16,17,18,19]. It has been shown that the number of TH neurons increases after DAergic denervation of the striatum due to a change in the phenotype of existing striatal neurons, and not due to neurogenesis [10,20]. These data are considered a manifestation of compensatory processes in response to the degradation of the nigrostriatal DAergic system. In this context, the most intriguing question is whether TH neurons are involved in compensatory DA synthesis [12]. However, all attempts to show that striatal neurons fully express the DAergic phenotype and synthesize DA in intact mammals have been unsuccessful [12]. Indeed, immunocytochemical data on the expression of aromatic L-amino acid decarboxylase (AADC, the second enzyme of DA synthesis), dopamine transporter (DAT), and vesicular monoamine transporter (VMAT) 2 in TH-expressing neurons in the striatum are controversial [6,10,14,20,21].

A powerful stimulus for studying the phenotype of TH-containing striatal neurons was the development of transgenic mice co-expressing green fluorescent protein (GFP) under the TH gene promoter. It was shown that these animals contain several thousand TH-expressing neurons [19,22]. When studying the wild-type and transgenic animals, convincing evidence was obtained that a significant part of TH neurons are gamma amino butyric acid (GABA)ergic neurons, the main structural and functional unit in the striatum [10,17,23,24]. However, in most neurons expressing the TH gene, the TH protein was not detected by immunocytochemistry [17,25]. It is believed that these neurons synthesize TH, but its content is beyond the resolution of immunocytochemistry [24]. Other protein markers of the DAergic phenotype, AADC, DAT, and VMAT2, are also undetectable with immunocytochemistry in striatal neurons of transgenic mice [21,25].

It follows from the above data that the question of the phenotype of striatal neurons expressing the TH gene and their ability to synthesize dopamine remains open. At the same time, it is tempting to elucidate whether there is a neural or neurohormonal regulation of striatal neurons partially expressing DAergic phenotype. Although such studies started quite recently, encouraging results have already been obtained, indicating that signaling molecules such as glutamate, DA, acetylcholine, and growth factors are involved in the regulation of striatal TH neurons [24,26]. The goal of this study was to determine to what extent striatal neurons express the DAergic phenotype and receptors for physiologically active substances and whether they are capable of synthesizing DA.

## 2. Results

### 2.1. Fluorescence Microscopy of Striatal Neurons Expressing Green Fluorescence Protein

**Figure 1:**
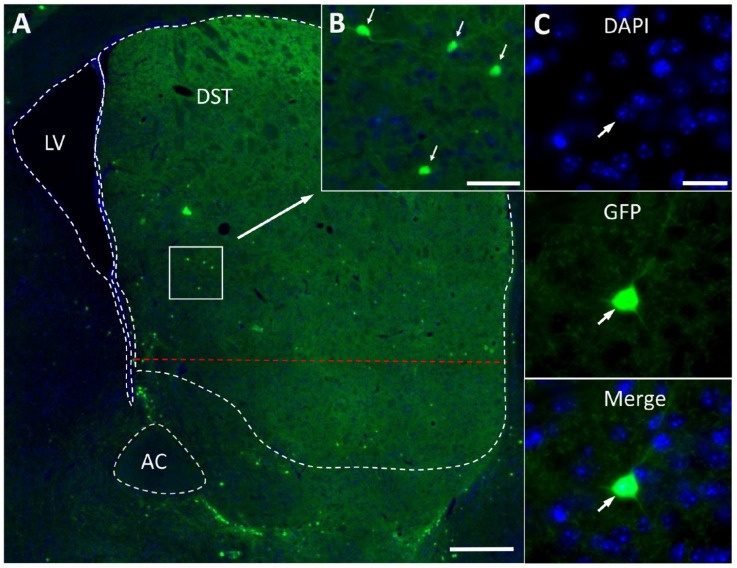
Green fluorescent protein (GFP)-expressing neurons in the frontal section of the dorsal striatum (DST) of transgenic mice. (**A**) General view of the DST. (**B**) Accumulation of GFP-expressing neurons in the DST. (**C**) Multipolar GFP-expressing neuron with 4′,6-diamidino-2-phenylindole(DAPI)-stained nucleus (blue). White dotted line is the contour of DST, lateral ventricle, and anterior commissure; red dotted line is the ventral border of the DST, conventionally taken in this study. Short and medium arrows show GFP-expressing neurons (green); long arrow shows the same DST fragment with a cluster of GFP-expressing neurons at low and high magnifications. AC, anterior commissure; LV, lateral ventricle. Scale bars: 500 µm for (**A**); 50 µm for (**B**); 20 µm for (**C**).

In *B6.B6D2-Tg(Th-EGFP)21-31Koba* mice, GFP-containing neurons were found all through the striatum. The largest number of GFP neurons appears to be located in the dorsal striatum, particularly along the border with the ventral striatum (Figure 1A). Quantitative analysis showed that the dorsal striatum of each cerebral hemisphere contains 3039 ± 258 GFP neurons (hereafter presented as mean ± SEM). These are round or oval bipolar or multipolar neurons with an average diameter of 11.74 ± 0.37 µm (Figure 1B,C).

**Figure 2:**
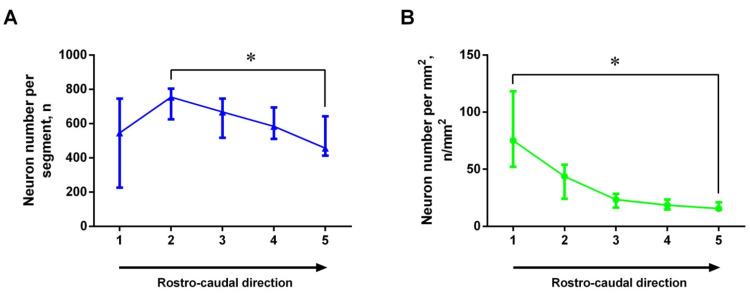
Change in the total number (**A**) and density (**B**) of green fluorescent protein (GFP)-expressing neurons in the frontal five segments of the dorsal striatum (each 320 μm long) along its rostro-caudal direction in transgenic mice (*n* = 4). * *p* < 0.05 (Friedman test with Dunn’s post-hoc test). Data are presented as median with interquartile range.

The number of GFP-expressing neurons per segment of the 320 μm long dorsal striatum in the rostro-caudal direction changes in such a way that the number of neurons in the second rostral segment is significantly greater than in the most caudal fifth segment (Figure 2A). As for the distribution density of GFP-expressing neurons (number of neurons per mm^2^), this parameter was higher in the rostral part of the dorsal striatum than in the caudal one (Figure 2B).

### 2.2. Confocal Microscopy of the Striatum of Transgenic Mice after Immunostaining for Tyrosine Hydroxylase and Aromatic L-Amino Acids Decarboxylase

**Figure 3:**
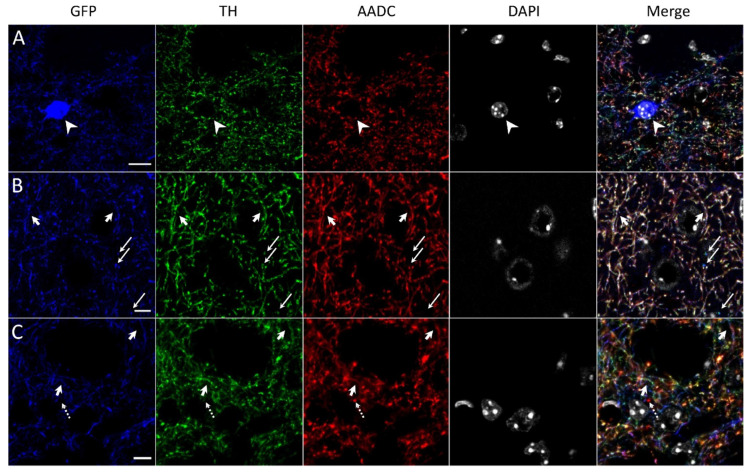
Confocal microscopy of the dorsal striatum of transgenic mice. (**A**) Neuron cell body containing green fluorescent protein (GFP, blue) and stained for 4′,6-diamidino-2-phenylindole (gray) (DAPI) but immunonegative for tyrosine hydroxylase (TH, green) and aromatic L-amino acid decarboxylase (AADC, red) (arrowhead). (**B**) Nerve fibers stained for GFP (blue), TH (green), and AADC (red) (short arrow), or for GFP and TH (long arrow). (**C**) Nerve fibers stained for GFP (blue), TH (green), and AADC (red) (short arrow), or for AADC (dotted arrow). Scale bars: 10 µm for (**A**); 5 µm for (**B**,**C**).

**Figure 4:**
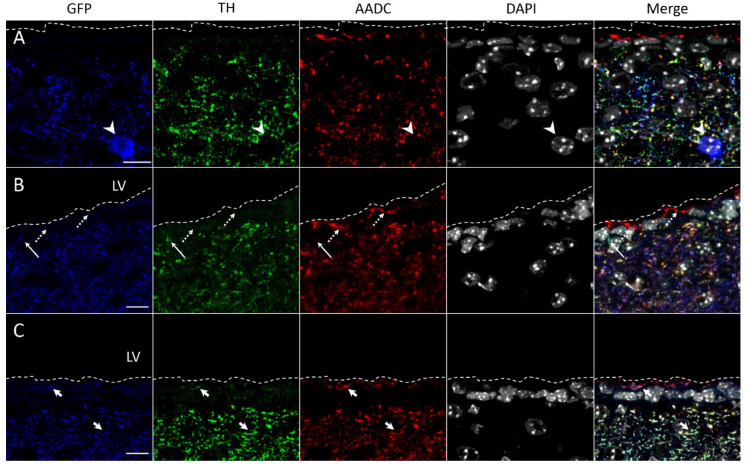
Confocal microscopy of supraependymal and periventricular areas of the dorsal striatum of transgenic mice. (**A**) The neuronal cell body containing green fluorescent protein (GFP, blue) and stained with 4′,6-diamidino-2-phenylindole (DAPI) (gray) but immunonegative for tyrosine hydroxylase (TH, green) and aromatic L-amino acid decarboxylase (AADC, red) (arrowhead). (**B**) Supraependymal nerve fiber stained for AADC (red) but lacking GFP (blue) and TH (green) (dotted arrow) and nerve fibers between ependymal cells stained for GFP and TH but immunonegative for AADC (long arrow). (**C**) Supraependymal and subependymal nerve fibers stained for GFP (blue), TH (green), and AADC (red) (short arrow). Scale bars: 10 µm for (**A**–**C**). LV, lateral ventricle. Dotted line is the apical surface of the ependymal lining.

Using double immunolabeling, we failed to detect TH- and AADC-immunopositive materials in GFP-expressing neurons in the striatum of transgenic mice (Figure 3A and Figure 4A). At the same time, nerve fibers containing GFP and immunopositive for TH (Figure 3B and Figure 4B) and for TH and AADC (Figure 3B,C and Figure 4B) were observed. In addition, AADC-immunopositive nerve fibers lacking GFP and TH-immunopositive material were found (Figure 3C and Figure 4C).

It should be noted that GFP-containing neuronal cell bodies and all the above-described types of nerve fibers in terms of the content of GFP, TH-, and AADC-immunopositive materials are contained in the entire striatum, including the periventricular region 200–300 μm wide along the ependymal wall of the lateral ventricles (Figure 4). Of particular interest are our observations of these nerve fibers located between ependymal cells and on their apical surface facing the cerebrospinal fluid. The former are represented by nerve fibers containing GFP, TH-, and AADC-immunopositive material or GFP and TH-immunopositive material (Figure 4C). The latter are represented by nerve fibers containing GFP, TH-, and AADC-immunopositive material or only AADC-immunopositive material (Figure 4B,C).

### 2.3. Obtaining a Fraction of GFP Neurons from the Dissociated Dorsal Striatum

**Figure 5:**
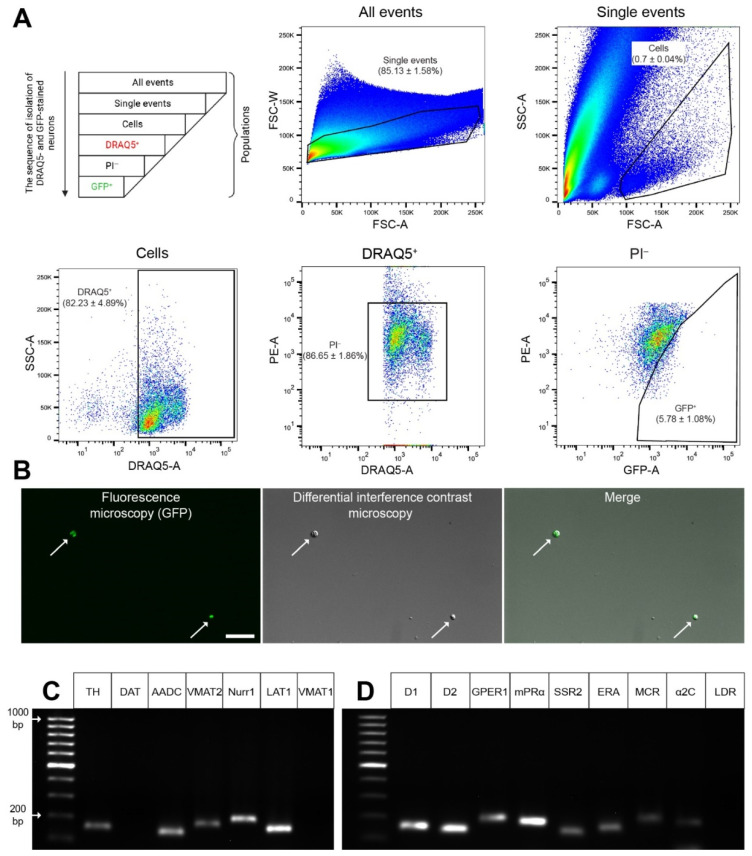
Neurons containing green fluorescent protein (GFP) isolated by fluorescence-activated cell sorting from a cell suspension of the dorsal striatum of transgenic mice, followed by assessment of the expression of specific genes in these neurons. (**A**) Characteristics of an isolated population of neurons stained with DRAQ5 (nuclear dye) and GFP. Histograms were plotted according to the density of all detected events. For each cell suspension, at least 1,000,000 events were analyzed. For each plot, *n* = 3. Data are presented as mean ± SEM. FSC, forward scatter; PE, phycoerythrin (detection channel); PI, propidium iodide; SSC, side scatter. (**B**) Fluorescent and differential interference contrast microscopy of isolated GFP neurons. Arrows show GFP-containing neurons. Scale bar: 50 µm. (**C**) Expression of genes for dopaminergic and non-dopaminergic phenotypes. (**D**) Expression of genes for neurotransmitter and hormone receptors. The leftmost lanes represent the DNA ladder (**C**,**D**). AADC, aromatic L-amino acid decarboxylase; α2C, alpha-2C adrenoceptor; D1, dopamine receptor 1; D2, dopamine receptors 2; DAT, dopamine transporter; ERA, endothelin receptor A; GPER1, G-protein coupled estrogen receptor 1; LAT1, L-amino acid transporter 1; LDR, L-DOPA receptor; MCR, mineralocorticosterone receptor; mPRα, membrane progesterone receptor alpha; Nurr1, nuclear receptor-related 1; SSR2, somatostatin receptor 2; TH, tyrosine hydroxylase; VMAT1, vesicular monoamine transporter 1; VMAT2, vesicular monoamine transporter 2.

In cell suspension of the striatum, we found individual events (individual cells and their individual fragments) and conglomerates, identified by forward and side scatter (see Section 4.7). When all events are taken as 100%, the individual events accounted for 85.13%. In turn, if all individual events are taken as 100%, individual cells (without fragments) amounted to 0.7%. If the isolated cells are taken as 100%, the cells with a nucleus stained with DRAQ5 amounted to 82.23%. Among the cells with a DRAQ5-stained nucleus, taken as 100%, living cells (unstained with propidium iodide (PI)) accounted for 86.65%. Among living cells, taken as 100%, GFP neurons accounted for 5.78% (Figure 5A). Thus, each sample prepared for analysis contained 3484 ± 417 living neurons expressing GFP and stained with DRAQ5.

According to microscopy, living GFP-stained cells sorted from the cell suspension of the dorsal striatum are spherical in shape due to the loss of their processes (Figure 5B). This indicates a homogeneous composition of the sorted cell population, which makes it possible to proceed to the assessment of gene expression in sorted GFP-stained neurons.

**Figure 6:**
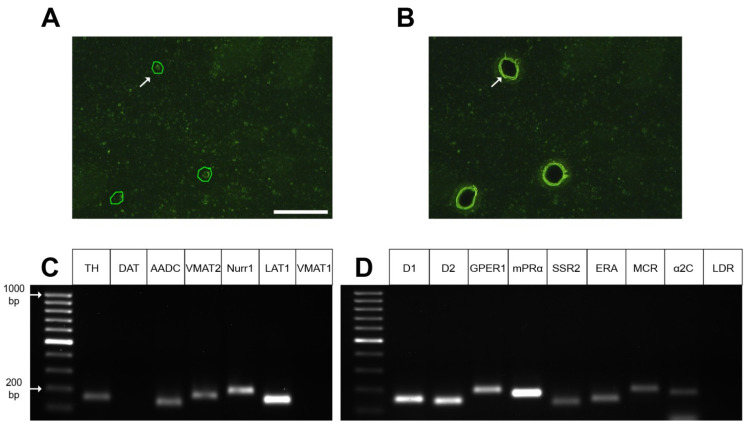
Laser microdissection of green fluorescence protein(GFP)-containing neurons from sections of the dorsal striatum of transgenic mice (**A,B**) followed by polymerase chain reaction analysis of the expression of genes for the dopaminergic phenotype (**C**) and receptors for signaling molecules (**D**). Scale bar: 50 µm for (**A**,**B**). Arrow shows outlines (green) of a striatal GFP neuron (**A**) and a hole after its laser microdissection (**B**). The leftmost lanes represent the DNA ladder (**C**,**D**). AADC, aromatic L-amino acid decarboxylase; α2C, alpha-2C adrenoceptor; D1, dopamine receptor 1; D2, dopamine receptor 2; DAT, dopamine transporter; ERA, endothelin receptor A; GPER1, G-protein coupled estrogen receptor 1; LAT1, L-amino acid transporter 1; LDR, L-DOPA receptor; MCR, mineralocorticosterone receptor; mPRα, membrane progesterone receptor alpha; Nurr1, nuclear receptor-related 1; SSR2, somatostatin receptor 2; TH, tyrosine hydroxylase; VMAT1, vesicular monoamine transporter 1; VMAT2, vesicular monoamine transporter 2.

Using laser microdissection, three samples for polymerase chain reaction (PCR) were prepared, each containing 1320 ± 7 striatal GFP neurons. Striatum obtained from two transgenic mice were used to prepare each sample (Figure 6A,B).

### 2.4. Gene Expression Profile in GFP-Containing Neurons of the Dorsal Striatum

Using PCR, it was found that the fraction of DRAQ5- and GFP-stained neurons isolated from the cell suspension of the dorsal striatum of transgenic mice (*B6.B6D2-Tg(Th-EGFP)21-31Koba*) is characterized by the expression of the genes for the following functionally important proteins: TH, AADC, VMAT2, nuclear receptor-related 1, L-amino acid transporter 1 (LAT1) (Figure 5C). However, they did not express genes for a DAT and VMAT1 (Figure 5C). In the same neurons, we found the expression of genes for DA receptor 1, DA receptor 2, endothelin receptor A, G-protein coupled estrogen receptor 1, membrane progesterone receptor alpha, somatostatin receptor 2, mineralocorticosterone receptor, and alpha-2C-adrenceptor. However, L-3,4-dihydroxyphenylalanine (L-DOPA) receptor mRNA expression was not detected (Figure 5D). Similar results were obtained with PCR in the study of individual GFP neurons excised from sections of the striatum of transgenic mice using laser microdissection. Indeed, we found in isolated GFP neurons gene expression for a number of proteins characteristic of DAergic neurons: TH, AADC, nuclear receptor-related 1, VMAT2, and LAT1 (Figure 6C). However, expression of the DAT and VMAT1 genes was not detected (Figure 6C). In addition, gene expression of all studied receptors was detected except for the L-DOPA receptor gene (Figure 6D).

### 2.5. Incubation of Striatal Sections with and without 2-Amino-2-Norbornanecarboxylic Acid

**Figure 7:**
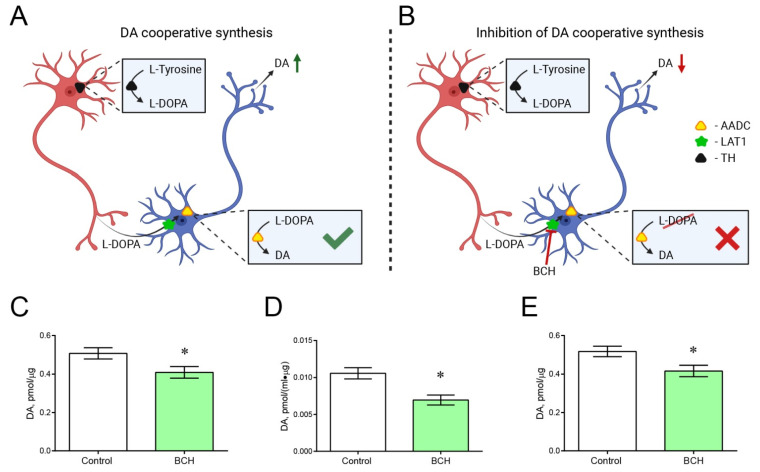
Testing the hypothesis of dopamine (DA) synthesis in transgenic mice by striatal neurons expressing one of the DA-synthesizing enzymes—tyrosine hydroxylase (TH) or aromatic L-amino acid decarboxylase (AADC). (**A**,**B**) Schematic representation of cooperative synthesis of DA by monoenzymatic neurons (**A**) and its inhibition by 2-aminobicyclo[2.2.1]heptane-2-carboxylic acid (BCH), an inhibitor of L-amino acid transporter 1 (LAT1) (**B**). (**C**) DA concentration in striatal sections of transgenic mice after one hour of incubation of sections in modified Krebs–Ringer solution with or without BCH. (**D**) DA concentration in the incubation medium after incubation of striatal sections of transgenic mice in Krebs–Ringer solution with or without BCH, normalized to the volume of the incubation medium and the protein concentration in striatal sections. (**E**) Total DA concentration in striatal sections of transgenic mice and the incubation medium after one hour of incubation of sections in Krebs–Ringer solution with or without BCH. * *p* < 0.05, significant difference (paired *t*-test). *n* = 9. Data are presented as mean ± SEM.

The previously developed method [27] was used to determine the hypothetical contribution of cooperative DA synthesis to the total DA content in the striatum of transgenic mice (Figure 7A,B). The concentration of DA in striatal sections obtained from transgenic mice was 19.3% lower after incubation with 2-aminobicyclo[2.2.1]heptane-2-carboxylic acid (BCH, Sigma-Aldrich, St. Louis, MO, USA) than after incubation without BCH (*p* = 0.0171) (Figure 7C). In the same experiment, the average concentration of DA in the incubation medium was 38.3% lower after incubation of striatal sections with BCH than in its absence, but this difference was not statistically significant (*p* = 0.1429). However, when the DA content in the incubation medium is normalized to the protein concentration in sections of the striatum, which is more correct, the difference between the DA concentration in the incubation medium following incubation of the striatal sections with and without BCH becomes statistically significant (*p* = 0.0122) (Figure 7D). Nonetheless, the total concentration of DA in striatal sections and in the incubation medium was 19.5% less after incubation of sections with BCH than after incubation without BCH (*p* = 0.0092) (Figure 7E).

## 3. Discussion

The objectives of this study were to elucidate (i) to what extent striatal neurons express the DAergic phenotype, (ii) what is the functional significance of striatal neurons expressing DA-synthesizing enzymes, and (iii) which intercellular signals can be involved in the regulation of these neurons. According to our data, (i) striatal neurons express DA-synthesizing enzymes—one of them (TH or AADC) or both; (ii) striatal neurons expressing individual enzymes of DA synthesis, TH or AADC, synthesize DA in cooperation; (iii) in addition to DA-synthesizing enzymes, striatal neurons contain VMAT2 but lack DAT, suggesting that they are not DAergic; (iv) nerve fibers containing DA-synthesizing enzymes project to lateral ventricles, providing a pathway for delivery of their secretory products (DA and L-DOPA) to the lateral ventricles; and (v) striatal neurons partially expressing the DAergic phenotype co-express receptors for a wide range of intercellular signals, suggesting their complex regulation.

Striatal neurons have long attracted the attention of researchers, since, on the one hand, they are involved in the control of motor behavior, and, on the other, they are regulated by nigral dopaminergic neurons [28]. The main targets for DA derived from nigral DAergic neurons are striatal GABAergic neurons, which provide the selection and execution of optimized motor sequences [29,30]. Interest in striatal GABAergic neurons has increased significantly after the discovery that some of them express the TH gene [1,8,14,17,21,31,32]. Moreover, as follows from immunocytochemical studies, the striatum of wild-type rodents contains rare neurons expressing AADC [33].

Although striatal neurons containing DA-synthesizing enzymes have long been detected by immunostaining, researchers are far from fully understanding their chemical phenotype and functional role [11,12,34]. Furthermore, they are faced with the problem of choosing the object for their study. The most accessible and widespread object—rodents—is unfavorable, since their striatum normally contains no more than 20 neurons that are immunopositive to DA-synthesizing enzymes. Although the number of such neurons is many times greater in the striatum of non-human primates, their use is limited for financial and ethical reasons [11,12]. Studies of striatal neurons expressing DA-synthesizing enzymes received a new impetus after it was shown that their number increased significantly after DAergic deafferentation of the striatum in neurotoxic animal models of Parkinson’s disease [11,12,18,19,20,33,35,36]. Even more promising has been the development of transgenic mice expressing GFP under the TH gene promoter using genetic constructs based on bacterial artificial chromosomes or a plasmid vector [17,22,25]. The striatum of each hemisphere of the brain in transgenic mice of the first type contains about 2700 neurons expressing the TH gene [22,32]. These data are consistent with our finding using transgenic mice of the second type [37,38]. In fact, we have shown that only in the rostro-dorsal striatum of one brain hemisphere in these transgenic mice, there are 3039 neurons expressing the TH gene.

Surprisingly, despite the presence of numerous neurons expressing the TH gene in the striatum of transgenic mice, neither our group nor previous researchers [25] have been able to detect TH-immunopositive material in these neurons. This is also the case for the striatum of intact wild-type C57BL/6 mice [36]. In contrast to the GFP-expressing neuron cell bodies, the vast majority of GFP-containing striatal fibers are immunopositive for both enzymes of DA synthesis or, much less frequently, for TH only or for TH and AADC. Interestingly, only rare GFP-containing nerve fibers are immunonegative for TH (see Section 2, Results). In addition, the striatum of transgenic mice contains fibers that are immunopositive for AADC, but lack GFP and TH-immunopositive material.

There is no doubt that the majority of striatal nerve fibers containing GFP and immunopositive for TH and AADC are axonal projections of nigral DAergic neurons. As for the origin of nerve fibers containing one of the DA-synthesizing enzymes, most of them belong to internal striatal neurons, which was shown in transgenic mice [22] and wild-type C57BL/6 mice [36] using markers of axonal transport. It remains unclear why DA-synthesizing enzymes are immunostained in neuron processes, but not in cell bodies. This can be explained either by the fact that synthesized enzymes do not accumulate in the neuron cell body, and are immediately transported to their processes, or by a change in immunostaining of the enzymes during their transportation. The first assumption seems more probable, since, in contrast to adult transgenic mice, in transgenic newborns with less developed neuron processes, TH-immunopositive material has been detected not only in neuron processes, but also in cell bodies [17].

Previous studies have emphasized that L-DOPA, as the final secretory product in monoenzymatic TH neurons, can play the role of a modulator of DA and norepinephrine receptors, as well as a neurotransmitter affecting specific receptors [39,40]. It is also suggested that L-DOPA controls the development of the brain as a morphogenetic factor [12,41]. Our knowledge of the functional role of L-DOPA can be expanded if taking into account our confocal microscopic observations of GFP-stained monoenzymatic TH-immunopositive nerve fibers located in the periventricular zone of the striatum and between ependymal cells of lateral ventricles. These data suggest that striatal monoenzymatic TH nerve fibers are one of the sources of L-DOPA contained in the cerebrospinal fluid [41].

Like monoenzymatic TH fibers, monoenzymatic AADC-immunopositive but GFP-lacking fibers have also been found in close proximity to the lateral ventricles. These fibers were found in the periventricular zone, between ependymal cells and on their apical surface, showing their penetration from the periventricular zone of the striatum into the lateral ventricles. Since DA is considered to be the final secretory product not only in DAergic neurons but also in monoenzymatic AADC neurons [40], our morphological observations indicate that DA could be delivered from monoenzymatic AADC fibers to the cerebrospinal fluid. This assumption is supported by the detection of DA in the cerebrospinal fluid in intact rats [42]. In addition to monoenzymatic nerve fibers, we found GFP bienzymatic fibers located in the periventricular zone of the striatum and on the apical surface of ependymal cells. These nerve fibers may belong to either nigral DAergic neurons or striatal bienzymatic non-DAergic neurons, being in any case a potential source of DA contained in the cerebrospinal fluid [42]. It can be assumed that L-DOPA and DA contained in the cerebrospinal fluid are involved in the regulation of brain neurons located in the periventricular region via volume neurotransmission. However, this hypothesis requires further evidence.

Since the initial demonstration that striatal neurons manifested some hallmarks of the DAergic phenotype, it became necessary to characterize their phenotype in more detail. This would clarify whether striatal neurons along with nigral DAergic neurons can participate in the DA regulation of motor behavior. Immunocytochemical studies addressing this issue have shown that DAergic deafferentation of the striatum of transgenic and C57BL/6 wild-type mice results in the appearance of neuron cell bodies and an increase in the number of nerve fibers immunopositive for TH only, AADC only, or both enzymes [18,19,20,21,33,35,36]. However, it has remained unclear whether VMAT2 and DAT, responsible for DA storage and DA uptake, respectively, are expressed in monoenzymatic and bienzymatic non-DAergic neurons.

To obtain comprehensive data on the gene expression of functionally important proteins in striatal neurons expressing the TH gene in transgenic mice, we first developed a method for isolating a fraction of GFP-containing neurons from the striatum of these animals. The fraction of neurons expressing the TH gene was isolated from the striatal cell suspension by sorting cells double-labeled for GFP and DRAQ5, a nuclear dye. Subsequent PCR analysis of gene expression for proteins of the DAergic phenotype has shown that the isolated cells express the TH, AADC, and VMAT2 genes, but do not express the DAT gene. The latter is consistent with the fact that it has been impossible to detect DAT-immunopositive material in striatal neurons expressing enzymes of DA synthesis [20], as well as with the resistance of these neurons to cytotoxins with high affinity for DAT [21].

To assess the gene expression of proteins of the DAergic phenotype and receptors for intercellular signaling molecules in striatal GFP neurons, along with the isolation of these neurons from the cell suspension of the striatum, laser microdissection of the same neurons from sections of the striatum was used. PCR data on the expression of genes of functionally important proteins, obtained in the study of GFP neurons isolated by laser microdissection, completely coincided with those obtained in the study of the fraction of GFP neurons selected from the cell suspension of the striatum.

Both approaches to the isolation of GFP neurons in the striatum in transgenic mice, which were used for the first time, are complementary and can serve for mutual validation. Each method has advantages and disadvantages. Indeed, when sorting GFP neurons from a cell suspension of the striatum, we obtain living cells that can be further used in experiments in vitro. In turn, laser microdissection of GFP neurons ensures greater preservation of mRNA, which allows minimizing the volume of samples for analysis. In addition, the isolation of cells using laser microdissection opens up the prospect of assessing the content of functionally important proteins with Western blot. The same study on GFP neurons obtained by sorting from a cell suspension of the striatum is problematic, since they are severely injured during the isolation process.

The data obtained in this study definitely show that there are no DAergic (DAT-expressing) neurons in the striatum of transgenic mice, whereas the isolated population of neurons can include neurons expressing only TH or both enzymes of DA synthesis. At the same time, the fact that some of the isolated striatal GFP-containing neurons express the VMAT2 gene responsible for DA storage in DAergic neurons [43] confirms our suggestion that striatal bienzymatic non-DAergic neurons are capable of storing DA.

One of the fundamental issues that we had to clarify was whether DA is synthesized by striatal non-DAergic monoenzymatic neurons. Indeed, after the immunocytochemical detection of TH in striatal neurons, it was suggested that they are capable of synthesizing DA [18,35]. However, for many years, this hypothesis could not be tested and had to be abandoned [21]. Later, we hypothesized and then obtained experimental evidence that the non-DAergic neurons of the arcuate nucleus, which express individual complementary enzymes of DA synthesis, TH or AADC, produce this neurotransmitter in cooperation [27,44]. In this case, L-DOPA, synthesized from L-tyrosine in monoenzymatic TH neurons, is released into the intercellular space and captured by LAT1 into nearby monoenzymatic AADC neurons, where DA is synthesized [11,27]. The evidence was based on the fact that upon incubation of tissue sections containing monoenzymatic neurons, DA synthesis is reduced in the presence of large molecules of neutral amino acids that are not involved in DA metabolism but compete with L-DOPA for LAT1 [45].

Using our methodology, in this study, vibratome sections of the dorsal striatum were incubated in the modified Krebs–Ringer solution containing the LAT1 inhibitor. In contrast to our previous research performed on vibratome sections of the arcuate nucleus [27], BCH was used as an inhibitor of LAT1 in this study. Unlike large neutral L-amino acids, BCH is a non-metabolizable inhibitor with a higher affinity for LAT1 [45]. As a control, vibratome sections were incubated in the Krebs–Ringer solution without BCH. The total content of DA in the nervous tissue and in the incubation medium after an hour of incubation was considered as an indicator of the level of DA synthesis in vibratome sections of the striatum. The presence of BCH in the incubation medium led to a decrease in the total DA content in vibratome sections and in the incubation medium, and, hence, in the DA synthesis compared to the control. This fact is considered as evidence of DA synthesis by monoenzymatic neurons in the striatum in transgenic mice.

When evaluating the functional role of monoenzymatic neurons in the striatum, as in other brain regions, it should be borne in mind that the secretion of L-DOPA and DA may not be the only function of these neurons. Indeed, almost all known monoenzymatic neurons synthesize non-monoamine neurotransmitters. Thus, TH is co-expressed with vasopressin or oxytocin in neurons of the supraoptic and paraventricular nuclei [46]. The second but not the last example is considered by the vasopressinergic neurons of the suprachiasmatic nucleus co-expressing AADC [47]. As shown in wild-type rodents and transgenic mice expressing GFP under the TH gene promoter, TH is colocalized with GABA in many, though not all, striatal GABAergic neurons [17,20,32,48]. In turn, more than 90% of GFP-stained neurons contain GABA in these mice [17]. In addition, along with neurons containing TH and GABA, neurons containing TH and enkephalin or dynorphin were found [17,20]. Although a large number of neurons co-express DA-synthesizing enzymes and non-monoamine neurotransmitters, it remains unclear whether these substances interact functionally. However, the only attempt to find a functional link between TH and kisspeptin co-expressed in neurons of the arcuate and periventricular nuclei of the hypothalamus was unsuccessful [49].

It is tempting to suggest that DA synthesized by mono- and bienzymatic non-DAergic neurons of the striatum is involved in the regulation of motor behavior together with DA synthesized in nigral DAergic neurons. In this regard, an increase in the number of striatal monoenzymatic neurons and activation of DA synthesis by these neurons, observed after DAergic denervation of the striatum in animals with specific neurotoxins (1-methyl-4-phenyl-1,2,3,6-tetrahydropyridin, 6-hydroxydopamine) and in humans with Parkinson’s disease [18,19,33,35,36], can be considered as a compensatory process initiated by the functional insufficiency of the nigrostriatal DAergic system. This implies the need to study the mechanisms of regulation of DA synthesis by striatal neurons as a platform for the development of a fundamentally novel antiparkinsonian therapy. There are already some data in the literature about the regulation of striatal mono- and bienzymatic non-DAergic neurons. These neurons were shown to be innervated by nerve fibers of various origins, for example, by axons of glutamatergic cortical neurons [24] or of nigral DAergic neurons [21]. In addition, striatal monoenzymatic TH neurons express receptors for acetylcholine [50,51].

We continued to study the regulation of striatal neurons partially expressing the DAergic phenotype by evaluating the expression of receptor genes for a number of physiologically active substances in an isolated population of GFP-containing neurons using PCR. We tested the gene expression of receptors for (i) classical neurotransmitters: DA (dopamine receptors, 1 and 2), norepinephrine (alpha-2C adrenoceptor), and L-DOPA; (ii) neuropeptides: somatostatin (somatostatin receptor 2) and endothelin (endothelin receptor A); and (iii) steroids: estrogens (G-protein coupled estrogen receptor 1), progesterone (membrane progesterone receptor alpha) and mineralocorticosterone. As a result, expression of all these receptor genes was detected in isolated striatal neurons, with the exception of the L-DOPA receptor gene. The latter seems to contradict the data, showing that in wild-type mice, exogenous L-DOPA increases the number of striatal TH-immunopositive neurons and the intensity of their immunostaining [20]. However, this seeming contradiction could be due to the fact that L-DOPA affects striatal neurons by modulating DA receptors 1, but not by its own receptors [52,53].

Our data on the expression of DA receptor 1 and DA receptor 2 genes in striatal neurons expressing the TH gene are consistent with previous data on the expression of these receptors in striatal neurons [23,26,54] and suggest that TH gene expressing neurons are regulated by nigral DAergic neurons [21]. It also cannot be excluded that there is an autoregulation of striatal monoenzymatic AADC neurons and bienzymatic neurons by DA, although this has not yet been tested.

As shown earlier, in addition to DA, norepinephrine is involved in the regulation of striatal neurons, acting through adrenoceptors, mainly of the α2C type [55,56,57], which are expressed in GABAergic neurons [58]. Our data obtained in transgenic mice provide direct evidence that α-2C-adrenoceptors are expressed in striatal neurons expressing the TH gene. Along with classical neurotransmitters such as catecholamines, serotonin, and acetylcholine, neuropeptides, particularly somatostatin, are involved in the regulation of the striatum [59,60]. Although the expression of the somatostatin gene in the striatum was described long ago [61], we were the first to discover that the somatostatin receptor 2 gene is co-expressed with the TH gene in striatal neurons.

Of particular interest is the regulation of neurons in the brain in general and in the striatum in particular by steroids that are synthesized in brain neurons and peripheral endocrine glands [62,63]. Steroids of peripheral origin are delivered to the brain from the general circulation, since there is no blood–brain barrier for them [64]. Indeed, it was shown that estrogens, particularly G-protein coupled estrogen receptor 1, whose gene expression we found in the isolated fraction of striatal TH gene-expressing neurons, has a morphogenetic effect on striatal neurons in the perinatal period of ontogenesis. This leads to sexual differentiation of the striatum [65].

In adulthood, estrogens have a modulatory effect on striatal neurons [64], particularly on GABAergic neurons [66]. According to Yoest et al. [67], they stimulate the release of DA from striatal neurons, and their action is modulated by progesterone. We have shown that isolated striatal GFP-containing neurons express, in addition to the TH and AADC genes, the estrogen and progesterone receptor genes required to control DA secretion. In this context, of particular interest is the study of Lemoine et al. [68], who showed with triple immunolabeling of TH, AADC, and progesterone receptors, that in the hypothalamic preoptic region, progesterone receptors are expressed only in AADC monoenzymatic neurons, whereas in the arcuate nucleus, these receptors are expressed in monoenzymatic TH neurons, in monoenzymatic AADC neurons, and in bienzymatic neurons.

The action of corticosteroids on striatal neurons is much less well understood than those of estrogen and progesterone. However, a number of papers showed that corticosteroids are involved in the stress response via striatum [69,70]. Of particular interest to us is the fact that corticosteroids control the release of dopamine in the striatum [71]. However, attempts to localize mineralocorticoid receptors in the striatum using immunological methods (Western blot, immunocytochemistry) were unsuccessful [72]. We were able to show for the first time that one of the targets for mineralocorticoids in the striatum are neurons expressing the TH gene.

Thus, we have obtained evidence that striatal neurons partially express the DAergic phenotype, synthesize DA, and are regulated by intercellular signals, which in sum opens broad prospects for the development of novel pharmacotherapy for the treatment of Parkinson’s disease.

## 4. Materials and Methods

### 4.1. Animals and Experimental Procedures

We used transgenic *B6.B6D2-Tg(Th-EGFP)21-31Koba* mice aged 8–12 weeks weighing 20–24 g (RIKEN BRC, Tsukuba-shi, Ibaraki, Japan) (*n* = 29) and *C57BL/6* mice (Laboratory Animal Farm Stolbovaya (SCBMT RAMS, Stolbovaya, Moscow reg., Russia)) (*n* = 2) of the same age and weight. The neurons of transgenic mice express the GFP gene under the TH promoter [73]. This animal line was maintained by crossing transgenic mice with inbred C57BL/6 mice. Transgenic mice were genotyped by amplification of tail tissue genomic DNA according to the RIKEN BRC protocol (Sheet ID PS_05058). The animals were kept at a temperature of 22 ± 1 °C, with a 12-hour day/night cycle and free access to food and water.

**Figure 8:**
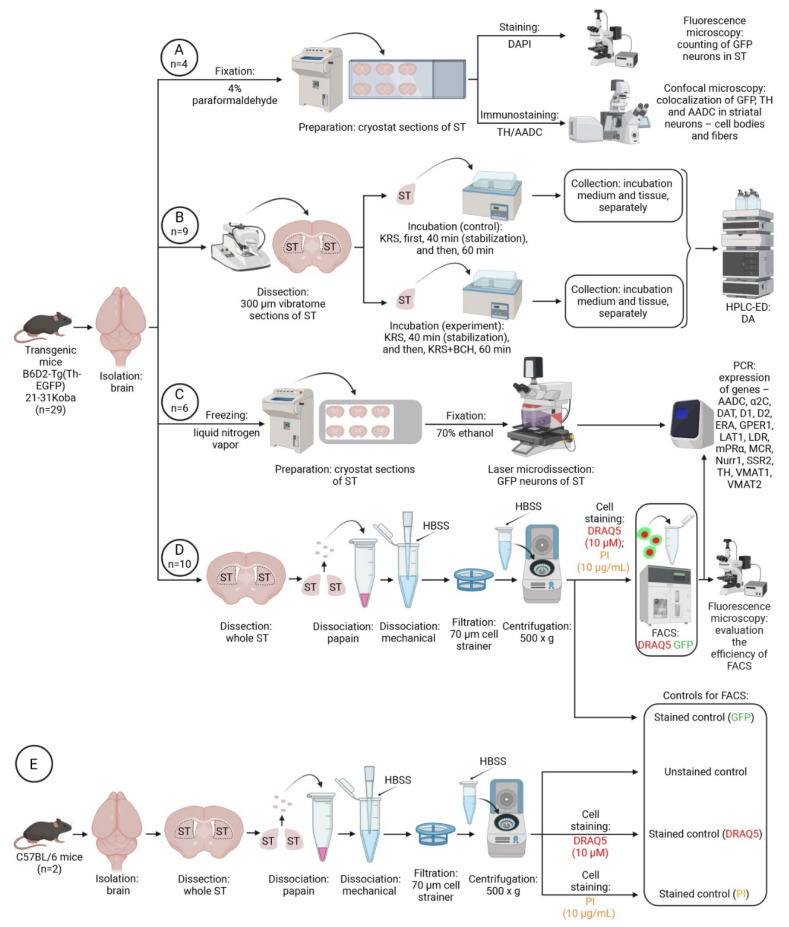
Design of experiments on the study of the striatum (ST) in transgenic mice *B6.B6D2-Tg(Th-EGFP)21-31Koba* and wild-type mice *C57BL/6*. (**A**) Morphological study of striatal neurons expressing one or more of the following proteins: green fluorescent protein (GFP), tyrosine hydroxylase (TH), and aromatic L-amino acid decarboxylase (AADC). (**B**) Assessment of cooperative synthesis of dopamine (DA) by monoenzymatic neurons expressing TH or AADC upon incubation of striatal sections in modified Krebs–Ringer solution (KRS) with (experiment) or without (control) 0.5 mM 2-aminobicyclo[2.2.1]heptane-2-carboxylic acid (BCH), L-amino acid transporter 1 (LAT1) inhibitor. (**C**) Isolation of GFP neurons by laser microdissection from striatal sections for subsequent analysis of the expression of functionally important genes. (**D**) Selection of the fraction of neurons stained with GFP (green) and DRAQ5 (red) from the cell suspension of the ST for subsequent analysis of the expression of functionally important genes. (**E**) Preparation of unstained striatal cell suspension, as well as striatal cell suspensions stained with DRAQ5 alone or with propidium iodide (PI) alone. α-2C, alpha-2C-adrenoceptor; D1, dopamine receptor 1; D2, dopamine receptor 2; DAPI, 4′,6-diamidino-2-phenylindole; DAT, dopamine transporter; ERA, endothelin receptor type A; FACS, fluorescence-activated cell sorting; GPER1, G protein-coupled estrogen receptor 1; HBSS; Hank’s Balanced Salt Solution; HPLC-ED, high-performance liquid chromatography with electrochemical detection; LDR, L-3,4-dihydroxyphenylalanine (L-DOPA) receptor; MCR, mineralocorticosterone receptor; mPRα, membrane progesterone receptor alpha; Nurr1, nuclear receptor-related 1; PCR, polymerase chain reaction; SSR2, somatostatin receptor 2; ST, striatum; VMAT1, vesicular monoamine transporter 1; VMAT2, vesicular monoamine transporter 2.

For immunohistochemical studies, the brains of some transgenic mice (*n* = 4) were isolated and frozen according to the previously described method [36,41]. Other transgenic mice (*n* = 25) were decapitated under anesthesia with 2.4% isoflurane (Baxter, Deerfield, IL, USA), and the brain was removed. The dissected dorsal striatum of these animals was used for ex vivo experiments (incubation of striatal vibratome sections), as well as to obtain a fraction of GFP neurons from the total cell suspension of the striatum and to isolate individual GFP neurons from striatal sections using laser microdissection (Figure 8). Along with transgenic mice, C57BL/6 mice were used. After decapitation of these animals under isoflurane anesthesia, a cell suspension was obtained from the striatum. Biological samples obtained after the incubation of vibratome sections were further analyzed by high-performance liquid chromatography with electrochemical detection and PCR.

### 4.2. Morphological Study

Using a cryostat (Leica CM1950, Leica Camera AG, Wetzlar, Germany), 20 µm thick frontal sections of the striatum were prepared from the frozen brains of transgenic mice at the coordinates 1.70 to 0.14 mm from Bregma in accordance with the mouse brain atlas [74] (Figure S1).

Every fourth cryostat section, starting from the first one, was mounted on a glass slide and placed into a medium containing 4’,6-diamidino-2-phenylindole (Abcam, Cambridge, UK). Every fourth cryostat section, starting from the second one, was mounted on a glass slide for subsequent immunostaining for TH and AADC (Figure 8A). For this, the sections were sequentially incubated in phosphate-buffered saline containing (i) 3% bovine serum albumin (Sigma-Aldrich, St. Louis, MO, USA), 0.3% Triton X-100 (Sigma-Aldrich, St. Louis, MO, USA), and 36 µL/mL Ig Blocking Reagent (M.O.M. Kit (Cat#FMK-2201, LOT#ZG0826), Vector Labs, Burlingame, CA, USA), for 1 h; (ii) 3% bovine serum albumin, 0.3% Triton X-100, and 80 µL/mL Protein Concentrate (M.O.M. Kit), for 20 min; (iii) mouse monoclonal antibodies to TH (1:700 (Cat#T1299, LOT#SLBY8300V), Sigma-Aldrich, St. Louis, MO, USA), 1% bovine serum albumin, and 0.1% Triton X-100, for 20 h; (iv) goat polyclonal antibodies to AADC (1:80 (Cat#AF3564, LOT#YOO0118081), R&D Systems, Inc. a Bio-Techne Brand, Minneapolis, MN, USA), phosphate-buffered saline, for 20 h; (v) biotinylated goat antibodies against mouse gamma globulins (1:200 (Cat#BA9200, LOT#T0206), Vector Labs, Burlingame, CA, USA) and 80 µL/mL Protein Concentrate (M.O.M. Kit), for 2 h; (vi) chicken Alexa Fluor 647 antibodies against goat gamma globulins (1:700 (Cat#A21469, LOT#2041655), Invitrogen, Thermo Fisher Scientific, Waltham, MA, USA) and 80 µL/mL Protein Concentrate, for 2 h; and (vii) CY3 conjugated with streptavidin (1:100 (Cat#S6402, LOT#050M6003), Sigma-Aldrich, St. Louis, MO, USA), for 60 min. All incubations were carried out at 20 °C. After each incubation, except the last one, the sections were washed three times in phosphate-buffered saline for a total of 45 min. After the last incubation, the sections were washed in phosphate-buffered saline for one hour and mounted into a medium containing 4′,6-diamidino-2-phenylindole.

### 4.3. Incubation of Striatal Sections of Transgenic Mice

The brain was obtained from mice decapitated under isoflurane anesthesia (*n* = 9). Four 300 µm thick frontal brain sections containing the striatum were prepared from each brain on a vibratome (Leica VT1200S, Leica Biosystems, Wetzlar, Germany) at 4 °C in Krebs–Ringer solution: NaCl 120 mM, KCl 4.8 mM, CaCl_2_ 2 mM, MgSO_4_ 1.3 mM, NaHCO_3_ 25 mM, D-glucose 10 mM, HEPES 20 mM, and ascorbic acid 0.1 mM (all from Sigma-Aldrich, St. Louis, MO, USA), pH = 7.2). Then, the striatum was excised from both hemispheres of the brain in a chilled Krebs–Ringer solution under a dissecting microscope (Leica M60, Leica Microsystems, Wetzlar, Germany).

For one sample, 4 sections of the striatum were dissected from one hemisphere of the brain from one animal. After that, the striatal sections were flow-incubated at a rate of 100 µL/min at 37 °C in thermostated chambers connected to a high-precision multichannel dispenser (Ismatec, Wertheim, Germany) (Figure 8B). Sections were first perfused in Krebs–Ringer solution for 40 min for functional stabilization, and then for 60 min in Krebs–Ringer solution containing 0.04% NH_4_OH (control) or in Krebs–Ringer solution containing 0.04% NH_4_OH and 0.5 mM BCH, LAT1 inhibitor (experiment) (Figure 8B). NH_4_OH was used to dissolve BCH [75]. After incubation, the medium was collected and 3,4-dihydroxybenzylamine hydrobromide was added at a final concentration of 10 nM, frozen in liquid nitrogen, and stored at −70 °C until high-performance liquid chromatography with electrochemical detection.

### 4.4. High-Performance Liquid Chromatography with Electrochemical Detection

High-performance liquid chromatography with electrochemical detection was used to determine DA in the incubation medium and in striatal sections after their incubation with and without BCH. Samples of the incubation medium were prepared by solid-phase extraction using aluminum oxide according to the protocol described earlier [36]. To measure DA in striatal sections, frozen tissue was homogenized with an ultrasonic homogenizer (Hielscher UP100H, Hielscher Ultrasonics GmbH, Teltow, Germany) at 4 °C in 400 µL of a solution of 0.1 M HClO_4_ and 250 pM/mL 3,4-dihydroxybenzylamine hydrobromide. Then, a portion of the homogenate was taken for protein concentration measurement using the BCA Protein Assay Kit (Thermo Fisher Scientific, Waltham, MA, USA) according to the manufacturer’s instructions. The rest of the homogenate was centrifuged at 18,000× *g* for 20 min at 4 °C, and the supernatant was collected for further analysis.

The determination of DA was carried out on a reverse phase column (100 × 4 mm ReproSil-Pur C18, 3 μm) (Dr. Maisch, Ammerbuch, Germany). The mobile phase was 0.1 M citrate–phosphate buffer containing 0.25 mM 1-octanesulfonic acid sodium salt, 0.1 M ethylenediaminetetraacetic acid, and 6% acetonitrile (pH = 2.55). The potential of substances leaving the column was determined using a DECADE II electrochemical detector (Antec Leyden, Leuden, The Netherlands). Peaks of catecholamines and their metabolites were identified by the release time relative to the standard solution. The content of DA was calculated using the ratio of peak areas in the sample to the standards. Peak areas were measured using LabSolutions software (Shimadzu, Kyoto, Japan). The content of DA was normalized to the total protein concentration.

### 4.5. Laser Microdissection

For laser microdissection, a piece of brain approximately 15 × 15 × 15 mm in size containing the rostro-dorsal striatum was excised. This piece of tissue was frozen in nitrogen vapor according to [76] and stored in tubes at −70 °C until further processing.

Frozen striatal samples were cut on a cryostat (Leica CM1950, Leica Biosystems, Wetzlar, Germany) into 12 µm sections, which were mounted on polyester membrane (FrameSlides POL-membranes 0.9 µm, Leica Biosystems, Wetzlar, Germany). Before cutting, the cryostat chamber, blade, working surfaces, and instruments were treated with RNAseClean solution (Biomedical Innovations, LLC, Rostov-on-Don, Russia) and 70% alcohol to protect mRNAs from degradation. Thirty-six sections were obtained from each sample of the striatum.

Sections mounted on POL membranes were fixed with 70% alcohol for 5 min at −20 °C and dried at room temperature for 15 min. After that, GFP cells were dissected using a laser microdissector (Leica LMD7000, Leica Microsystems, Wetzlar, Germany) at 40× magnification, laser power of 35 µJ, laser spot aperture of 5 µm, and laser pulse frequency of 1000 Hz (Figure 8C). Individual tubes containing 50 μL TRI Reagent (Sigma, St. Louis, MO, USA) and 100 U/mL RiboLock RNase inhibitor (Thermo Fisher Scientific, Waltham, MA, USA) were used to collect 650 cells from each striatum. Finally, the tubes were frozen in liquid nitrogen and stored at −70 °C until PCR. The description of the method for laser microdissection of cells from fixed tissue sections is given in more detail in previous papers [77,78].

### 4.6. Preparation of a Cell Suspension from the Dorsal Striatum of Transgenic Mice and C57BL/6 Mice

The dissociation of the striatum and the preparation of the cell suspension were carried out according to the modified protocol of Liu et al. [79]. Mice were decapitated under anesthesia, and the dorsal striatum was excised from the brain under a dissecting microscope. Striatal tissue of two brains was cut with razor blades into 1 mm^3^ pieces, which were pooled and transferred to foil-wrapped tubes containing 200 µL of chilled Dulbecco’s Modified Eagle Medium (Gibco, Thermo Fisher Scientific, Waltham, MA, USA) (Figure 8D). It should be noted that all solutions that were further used for fluorescence-activated cell sorting and PCR contained 100 U/mL of the RNase inhibitor RiboLock. Striatal tissue was precipitated by centrifugation at 425× *g* for 2 min at 4 °C (Eppendorf, Hamburg, Germany). The supernatant was collected and 200 µL of Dulbecco’s Modified Eagle Medium containing 2 mg/mL papain (Sigma-Aldrich, St. Louis, MO, USA) was added to the precipitate. Papain was pre-dissolved in Dulbecco’s Modified Eagle Medium and activated by incubation for 30 min at 37 °C. The precipitate was vortexed and incubated in papain solution for 30 min at 37 °C with constant stirring, which promotes the dissociation of the nervous tissue [80,81]. The enzymatic action of papain was stopped by adding chilled (4 °C) fetal bovine serum (Gibco, Thermo Fisher Scientific, Waltham, MA, USA) to the tubes at a final concentration of 10% (*v*/*v*). The resulting solution was stirred on a vortex. The tubes with nervous tissue were centrifuged at 425× *g* for 2 min at 4 °C, and the supernatant was collected. The resulting precipitate was resuspended in 400 µL Hank’s Balanced Salt Solution (Gibco, Thermo Fisher Scientific, Waltham, MA, USA) and vortexed. The tissue was reprecipitated and resuspended in 400 µL of chilled Hank’s Balanced Salt Solution according to the previously described protocol, and then reprecipitated for 2 min at 4 °C. The supernatant was removed; the precipitate was resuspended in 200 µL of chilled Hank’s Balanced Salt Solution and centrifuged at 425× *g*. The resulting solution was pipetted by passing the contents of the tube 20 times through a 1000 µL tip with a diameter of 1 mm. The tubes were in a vertical position on ice for 5–7 min, which led to the settling of undissociated pieces of tissue on the bottom of the tube. The supernatant—the cell suspension that did not settle on the bottom of the tube—was collected into another tube placed on ice. The remaining undissociated tissue was resuspended in 200 µL chilled Hank’s Balanced Salt Solution. Then, the tissue was re-dissociated by passing the contents of the tube 10 times through a 200 µL tip with a diameter of 0.5 mm. After that, the tubes were in a vertical position on ice for 5–7 min, which led to the precipitation of undissociated cell conglomerates. The supernatant was carefully collected and combined with the cell suspension selected in the previous step. The remaining undissociated tissue was resuspended in 200 µL of cold Hank’s Balanced Salt Solution, and the last step of dissociation and precipitation was repeated. The supernatant was combined with the previously selected suspensions. The resulting solution was stirred and filtered through a 70 µm cell strainer (Falcon, Corning, Corning, NY, USA) into another tube. The suspension was centrifuged at 500× *g* for 5 min at 4 °C. The supernatant was collected and the precipitate was resuspended at room temperature in 100 µL Hank’s Balanced Salt Solution containing 10 µM DRAQ5 (Abcam, Cambridge, UK), dye of nuclei of living and fixed cells [82]. The suspension was incubated for 5 min at 37 °C. The stained cell suspension was kept cold in foil-wrapped tubes until analyzed and sorted on a cell sorter. Prior to sorting, the suspension was adjusted to 500 µL with chilled Hank’s Balanced Salt Solution containing 10 µg/mL PI (Sigma-Aldrich, St. Louis, MO, USA), a stain for dead cell nuclei, and incubated for 5 min at 4 °C with shaking (Figure 8D).

### 4.7. Cell Sorting

Neurons obtained by dissociating the dorsal striatum of transgenic mice and stained with DRAQ5 and PI were sorted into microcentrifuge tubes on a FACSAria III cell sorter (BD, Franklin Lakes, NJ, USA) using a 100 µm nozzle at a pressure of 20–21 psi. The threshold staining levels for DRAQ5, GFP, and PI were determined by analyzing the unstained cell suspension obtained from wild-type mice and single stain controls: DRAQ5, GFP, and PI (Figure S2). DRAQ5 was excited with a 640 nm laser and detected with a 670/30 nm band pass filter (DRAQ5 channel), whereas GFP was excited with a 488 nm laser and detected with a 530/30 nm band pass filter (GFP channel). PI was excited with a 488 nm laser and detected with a 585/42 nm band pass filter (PI channel). Histograms were plotted according to the density of all detected events (all detected particles), and the results were analyzed using FlowJo software (BD, Franklin Lakes, NJ, USA). It should be noted that single events—individual cells or their individual fragments—differ from their conglomerates by forward and side scatter.

Four samples of sorted cell suspension were obtained, which were stained with GFP and DRAQ5 and unstained with PI. One sample was used to evaluate the efficiency of fluorescence-activated cell sorting under a fluorescence microscope. The remaining three samples were supplemented with 500 µL TRI Reagent containing 100 U/mL RiboLock, RNase inhibitor. These samples were frozen in liquid nitrogen and stored at −70 °C until PCR.

### 4.8. RNA Isolation and PCR

Isolation of total RNA from the sorted cells was performed by adding 500 µL of TRI Reagent to 500 µL of thawed TRI Reagent containing the sorted cells. Before extraction of total RNA from cells obtained by laser microdissection, the samples were thawed and the contents of two tubes (1300 cells) were used as one sample. Then, 900 µL of TRI Reagent was added to each sample. Further procedures for total RNA processing and synthesis of complementary DNA were the same both for the fraction of sorted cells and for individual cells obtained by microdissection.

The resulting samples were incubated in tubes at 20 °C for 5 min. Then, 100 µL of 1-bromo-3-chloropropane (Sigma-Aldrich, St. Louis, MO, USA) was added to each tube and the incubation continued at 20 °C for 15 min, vortexing the solution every 3 min. The tubes were centrifuged at 21,000× *g* for 15 min at 4 °C. The aqueous phase containing RNA was transferred to another microcentrifuge tube, and 500 µL isopropyl alcohol (Sigma-Aldrich, St. Louis, MO, USA) was added. For better RNA precipitation, 1 µL glycogen (Thermo Fisher Scientific, Waltham, MA, USA) was added. Samples were vortexed and incubated at 20 °C for 10 min, after which total RNA was precipitated by centrifugation at 21,000× *g* for 10 min at 4 °C. The supernatant was removed and the precipitate was washed three times in 1 mL 80% ethanol and centrifuged at 21,000× *g* for 10 min at 4 °C. After the last centrifugation, ethanol was removed and the RNA precipitate was air-dried for 15 min. Then, RNA was dissolved in 20 µL PCR-grade water (Evrogen, Moscow, Russia). The total RNA concentration in all samples was measured using a NanoDrop 8000 spectrophotometer (Thermo Fisher Scientific, Waltham, MA, USA). Residual genomic DNA was removed by DNase I (Thermo Fisher Scientific, Waltham, MA, USA) according to the manufacturer’s protocol.

Complementary DNA was synthesized from 100 ng total RNA using the Maxima H Minus First Strand cDNA Synthesis Kit (Thermo Fisher Scientific, Waltham, MA, USA) according to the manufacturer’s protocol. Complementary DNA concentration was measured using a NanoDrop 8000 spectrophotometer. PCR was performed using qPCRmix-HS SYBR+LowROX (Evrogen, Moscow, Russia) on a QuantStudio 12k Flex cycler (Applied Biosystems, MA, USA). In sorted cells, we assessed by PCR the expression of the following genes for neuronal dopaminergic phenotype proteins: TH, DAT, AADC, VMAT2, and nuclear receptor-related 1; genes for transporters: LAT1 and VMAT1. In addition, expression of the following receptors was assessed by PCR: alpha-2C adrenoceptor, DA receptor 1, DA receptor 2, endothelin receptor type A, G protein-coupled estrogen receptor 1, L-DOPA receptor, membrane progesterone receptor alpha, mineralocorticosterone receptor, and somatostatin receptor 2. Oligonucleotide primers were used to determine the expression of these genes (Table S1, Evrogen, Moscow, Russia). PCR was performed using 500 ng of complementary DNA and the following amplification protocol: hold stage at 50 °C for 2 min and then at 95 °C for 10 min; PCR stage at 95 °C for 15 s and then at 60 °C for 1 min (50 cycles); melt curve stage successively at 95 °C for 15 s, at 60 °C for 1 min, and at 95 °C for 15 s. After the end of the reaction, the amplified products were separated at a voltage of 100 V in 1.5% agarose gel (Helicon, Moscow, Russia) containing ethidium bromide (Thermo Fisher Scientific, Waltham, MA, USA). DNA fragments were detected using ChemiDoc Touch (Bio-Rad Laboratories, CA, USA).

### 4.9. Fluorescence Microscopy

Striatal GFP neurons were examined on sections using a Zeiss Axio Observer Z1 fluorescent microscope equipped with a Zeiss Colibri LED illumination system and a Zeiss AxioCam MRc camera (Zeiss, Oberkochen, Germany), with a 20× objective. We used 9 × 9 tiles with a 10% overlap to create panoramic images. Mosaic stitching was carried out using AxioVision 40 V 4.8.2.0 software (Zeiss, Oberkochen, Germany).

The diameter and area of the neuron cell bodies were determined on sections with the AxioVision 40 V 4.8.2.0 software using the line and outline tools, respectively. This analysis was performed on cells with a visible nucleus and axonal hillock. The neuron size was determined by drawing a line from the axonal hillock to the opposite edge of the cell.

For microscopy of living neurons stained with DRAQ5 and GFP, the sorted suspension was placed on a coverslip and covered with a round coverslip, 15 mm in diameter. The resulting preparations were studied under a Zeiss Axio Observer Z1 fluorescent microscope. Images were acquired at a 40× objective and analyzed using AxioVision 40 V 4.8.2.0 software. Living neuron microscopy was performed over the entire area of the coverslip, 176.7 mm^2^, analyzing images of all sorted cells.

### 4.10. Counting Cell Bodies of GFP Neurons in the Dorsal Striatum

The GFP neuron cell bodies were counted on every fourth frontal cryostat section of the dorsal striatum using the FiJi software (available online: (https://imagej.net/software/fiji/downloads) accessed on 10 August 2022) as follows: the images were converted to 8-bit and the dorsal striatum was outlined using the ROI for further analysis. The threshold fluorescence was set so that the fluorescence intensity of GFP neurons was higher than the fluorescence of unstained structures (autofluorescence). Then, using soft Analyze Practice, we counted all GFP-containing structures with an area larger than 10 µm^2^, which we consider to be neuronal cell bodies.

The total number of GFP neurons in the rostro-dorsal striatum was counted using a previously proposed method [83,84]. This makes it possible to estimate the total number of cells in a given volume of tissue by counting them in individual sections taken from a complete series of sections with a constant distance between the selected sections. Importantly, this method uses the Abercrombie test [85] to avoid double counting cells. To calculate the total number of GFP neurons in the rostro-dorsal striatum, we took every 4th section from the complete series of sections of this brain region and used the following formula:

$$N=\overset{i}{\underset{i=1}{\sum }}\left(ni\;\left(Si+1\right)\;\frac{h}{h+D}\right)$$

*N*, the total number of neurons;

*ni*, the number of neurons per selected section;

*Si*, number of skipped sections;

*h*, section thickness, in μm; and

*D*, neuron diameter, in μm.

In addition to the total number of GFP neurons in the dorsal striatum, the density of their distribution was assessed. For this, the area of the dorsal striatum was determined in each selected section, and the number of neurons per unit area (1 mm^2^) was calculated. These data make it possible to quantify the distribution of neurons in the striatum along the rostro-caudal direction.

### 4.11. Confocal Microscopy

To assess the colocalization of GFP, TH, and AADC, striatal sections were examined with a confocal microscope Zeiss LSM880 (Zeiss, Oberkochen, Germany) using the objectives Plan-apochromat 20 × 0.8 M2 and Plan-Apochromat 63×/1.40 Oil DIC M27. Photographs were taken in two channels. In the first channel, the signals from AlexaFluor 647 and GFP were simultaneously detected; in the second channel, those from CY3 and 4′,6-diamidino-2-phenylindole were simultaneously detected. To assess the topographic relationships between the labeled neurons (cell bodies) and nerve fibers, a z-stack was made (optical section thickness 0.5 μm at a magnification of 63× with an additional 2× zoom). Image processing was performed with a ZenBlue software (Zeiss, Oberkochen, Germany).

### 4.12. Statistics

Statistical analysis was performed using GraphPad Prism 6 software (GraphPad Software, La Jolla, CA, USA). The groups were compared for normality by using the Shapiro–Wilk test. For pairwise comparison, we used the paired t-test. For multiple comparisons, we used the Friedman test with Dunn’s post-hoc test. The results are presented as mean ± SEM or as median with interquartile range. Differences were considered significant at *p* < 0.05.

## Data Availability

The data presented in this study are available on request from the corresponding author. The data are not publicly available due to legal issues.

## Supplementary Materials

The following supporting information can be downloaded at: https://www.mdpi.com/article/10.3390/ijms231911054/s1

## Abbreviations

AADC	aromatic L-amino acid decarboxylase
BCH	2-aminobicyclo[2.2.1]heptane-2-carboxylic acid
DA	dopamine
DAT	dopamine transporter
GABA	gamma amino butyric acid
GFP	green fluorescent protein
LAT1	L-amino acid transporter 1
L-DOPA	L-3,4-dihydroxyphenylalanine
PCR	polymerase chain reaction
PI	propidium iodide
TH	tyrosine hydroxylase
VMAT	vesicular monoamine transporter
