# Supplementary material for: Striatal Neurons Partially Expressing a Dopaminergic Phenotype: Functional Significance and Regulation
Source: Int J Mol Sci. 2022 Sep 21;23(19):11054. doi: 10.3390/ijms231911054 (PMC9570204; doi:10.3390/ijms231911054)
Supplement: Supplementary file 1 [file ijms-23-11054-s001.zip › Table S1.pdf]

International Journal of
Molecular Sciences

MDPI

Table S1. Oligonucleotide primers used for PCR.

Gene designations by NCBI database	Protein	Forward Primer	Reverse Primer
Cyc1	Cytochrome C	5'-GCGGCCAGGGAAGTTGT-3′	5'-GCCAGTGAGCAGGGAAAATAC-3′
Th	Tyrosine hydroxylase	5'-TCAGAGGAGCCCGAGGTC-3'	5'-GGGCGCTGGATACGAGAG-3'
Slc6a3	Dopamine transporter	5'-CATCAACCCACCGCAGA-3'	5'-GAAGGCACCTCCACCATTT-3'
Ddc	Aromatic L-amino acide decarboxylase	5'-TCCCCACGGCTAGCTCATACCC-3'	5'-TTCCCCAGCCAGTCCATCATCA-3'
Drd1	Dopamine receptor 1	5'-CTTTGGAGAGGGCAGCAGT-3'	5'-ACCCCCATGATCACAGACAGT-3'
Drd2	Dopamine receptor 2	5′-GAACAGGCGGAGAATGGA-3′	5′-GGATGGATCGGGGAGAGT-3′
Nr4a2	Nuclear receptor related 1	5'-CCGAAGAGCCCACAGGAT-3'	5'-CCATAGAGCCGGTCAGGAG-3'
Slc18a2	Vesicular monoamine transporter 2	5'-ATTGGCTTTCCTTGGCTCAT-3'	5'-GGTACGGCTGGACATTATTCTG-3'
Slc18a1	Vesicular monoamine transporter 1	5'-CCGGAAGCTGGTGTTGGT-3′	5'-GAGAAGGCAGGAGAGGTGAGA-3′
Slc7a5	L-type amino acid transporter 1	5'-CTCCCGGTGTTCTTTATCCTG-3′	5'-AGAATCCACTTGGGCTTGTT-3′
Gper1	G protein-coupled estrogen receptor 1	5′-TCCGGGAGAAGATGACCA-3′	5′-GAGGAAGAAGACGCTGCTGTA-3′
Paqr7	Membrane progesterone receptor alpha	5′-GCGCTGGCCTATGTGCT-3′	5′-CTGCCGGGGAACCAACT-3′
Sstr2	Somatostatin receptor 2	5′-CATCCTGTACGCCTTCTTGTCT-3′	5′-TCATTCAGCCGGGATTTGT-3′
Ednra	Endothelin receptor type A	5′-GCCCACAGCAGACTAAAATCAC-3′	5′-TGGGGCCGTTCCTCATAC-3′
Adra2c	alpha 2C adrenoceptor	5′- CGTGTTCGTACTGTGCTGGTT-3′	5′-CGCCTGAAGTCCTGATTGAA-3′
Gpr143	G Protein-Coupled Receptor 143 – L-DOPA receptor	5′-CCCCAGGAAGGTTGTATGTGT-3′	5′-CTGGGCTTGGGAAATGGA-3′
Nr3c2	Nuclear receptor subfamily 3 group C member 2 – mineralocorticosterone receptor	5′-GGGGGATTAAGCAAGAACC-3′	5′-GACAGGCGGAAAGGAACTC-3′

NCBI, National Center for Biotechnology Information (https://www.ncbi.nlm.nih.gov/gene)
