# Supplementary figures and images for: Striatal Neurons Partially Expressing a Dopaminergic Phenotype: Functional Significance and Regulation
Source: Int J Mol Sci. 2022 Sep 21;23(19):11054. doi: 10.3390/ijms231911054 (PMC9570204; doi:10.3390/ijms231911054)

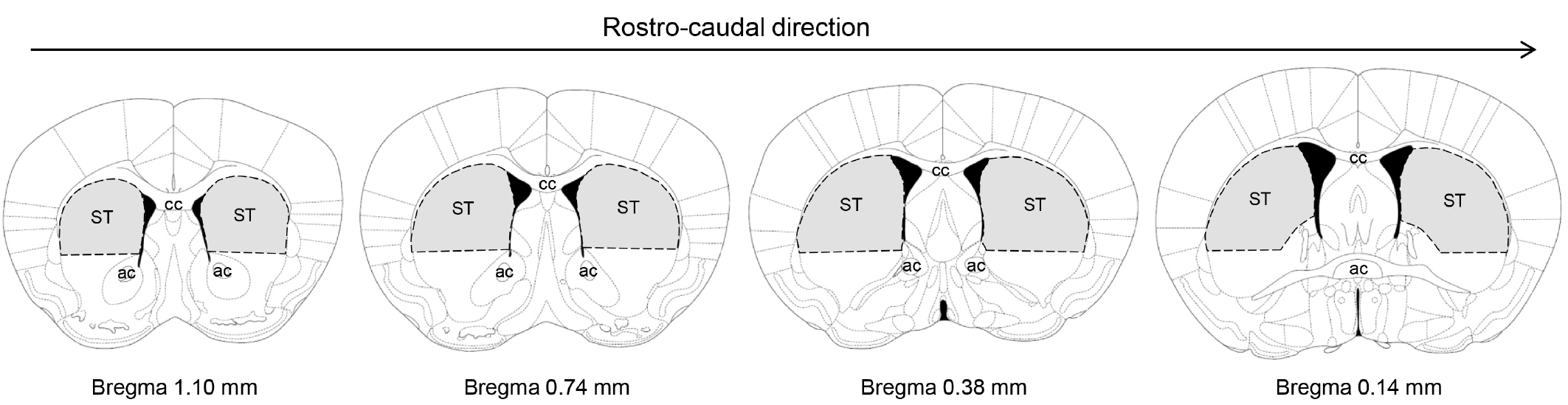

Supplement: Supplementary file 1 [file ijms-23-11054-s001.zip › Figure S1.jpg]

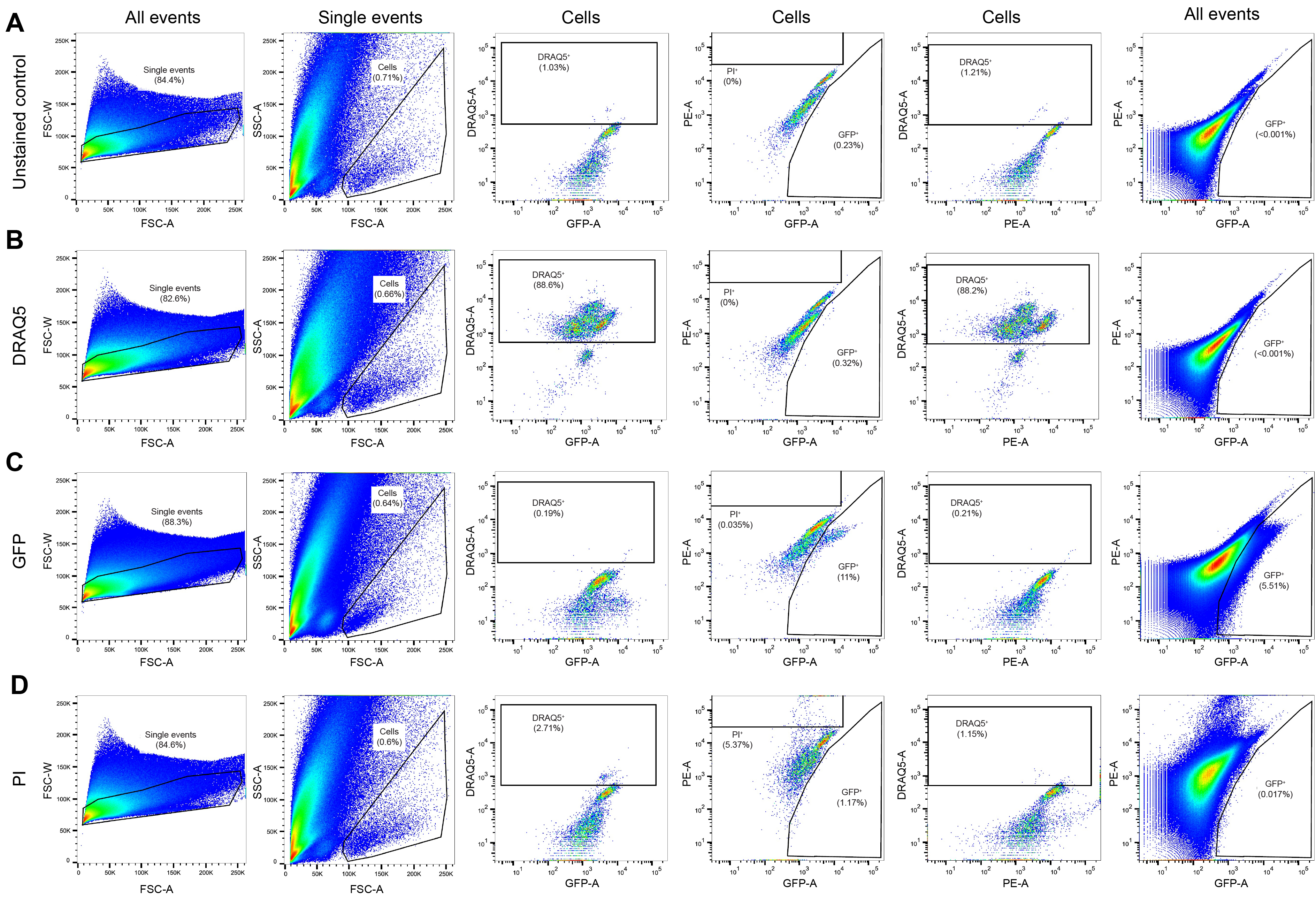

Supplement: Supplementary file 1 [file ijms-23-11054-s001.zip › Figure S2.jpg]
